# Integrating Subacute Ruminal Acidosis, Lipopolysaccharide, and Trained Immunity: A Comprehensive Review

**DOI:** 10.7150/ijbs.104074

**Published:** 2025-03-31

**Authors:** Guobin Hou, Jingjun Wang, Shuai Liu, Duo Gao, Yiming Xu, Yimin Zhuang, Wenzhuo Dong, Yi Yue, Jinni Bai, Shangru Li, Jiaying Ma, Mengmeng Li, Wei Wang, Yajing Wang, Shengli Li, Zhijun Cao

**Affiliations:** 1 State Key Laboratory of Animal Nutrition and Feeding, International Calf and Heifer Organization, College of Animal Science and Technology, China Agricultural University, Beijing, 100193, China.; 2 College of Animal Science and Technology, Beijing University of Agriculture, Beijing, 102206, China.; 3 College of Animal Science, Xinjiang Agricultural University, Urumqi, Xinjiang, 830052, China.

**Keywords:** subacute ruminal acidosis, ruminant, lipopolysaccharide, inflammation, trained immunity

## Abstract

Subacute ruminal acidosis (SARA) has emerged as a prevalent digestive disorder that significantly affects the overall health of ruminants, with notable links to various inflammatory diseases. Throughout the progression of SARA, elevated lipopolysaccharide (LPS) levels in the rumen play a crucial role in initiating the innate immune response. In this review, we evaluate the recent insights into the pathways associated with SARA-induced inflammatory responses, with a specific focus on LPS. It is important to recognize the variation in the immune response activation potential of LPS derived from different bacterial sources. This variability aligns with the widespread detection of LPS in the rumens of ruminants with SARA. Nonetheless, trained immunity is expected to become a novel strategy for the prevention and control of SARA. This mechanism offers a rapid response to secondary stimuli, including LPS, effectively preventing inflammation. Ultimately, this review establishes a comprehensive system integrating SARA, LPS, and trained immunity. Through this integrated approach, we aim to provide innovative solutions to the challenges associated with SARA.

## Introduction

High-quality dairy products play a vital role in human nutrition and disease prevention 1. Cows represent a significant source of dairy products. High-concentrate (HC) diets are employed in the agricultural industry to improve the efficiency of dairy production. However, these diets contain fermentable carbohydrates, which promote the production and accumulation of volatile fatty acids (VFAs) in the rumen, causing a decrease in rumen pH and leading to subacute ruminal acidosis (SARA). SARA can be considered to occur when pH levels are below 5.6 or 5.8 for a minimum of 3 or 5.4 hours/day, respectively 2,3. Although ruminants can neutralize excessive VFAs by secreting saliva, the low proportion of neutral detergent fiber hinders the production of sufficient saliva to effectively counteract the negative effects of VFAs 4. This prolonged reduction in pH distinguishes SARA from acute ruminal acidosis caused by lactic acid accumulation 5,6. Cows experiencing acute rumen acidosis exhibit a rumen pH below 5.0, as well as increased lactic acid in the rumen and blood, an increased abundance of gram-positive and lactic acid-producing bacteria 7.

**Figure 1:**
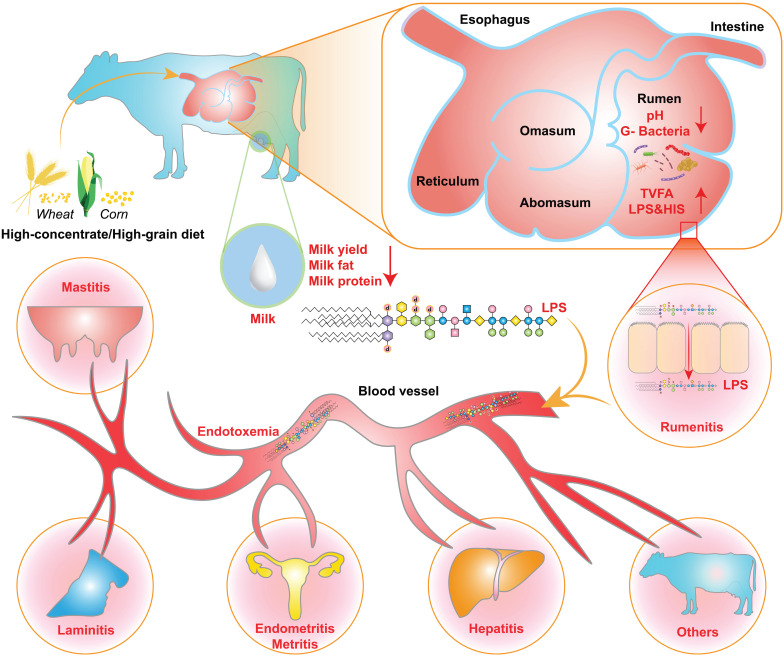
** SARA-induced inflammatory disease mechanisms.** Prolonged consumption of high-concentrate diets disrupts the internal rumen environment in dairy cows, characterized by the accumulation of TVFA, LPS, and HIS, along with a decrease in ruminal pH, milk yield, milk fat and protein, and the lysis of Gram-negative bacteria. These changes exert persistent deleterious effects on rumen epithelial cells, including the disruption of tight junctions, which facilitates the translocation of LPS into systemic circulation. Once LPS reaches various tissues and organs and accumulates beyond a critical threshold, it has the potential to trigger inflammatory responses.

The increase in the ruminal concentration of lipopolysaccharide (LPS) has been well documented for SARA challenges based on HC diets. The change in ruminal conditions caused by these challenges results in lysis and death of gram-negative bacteria, and leads to the release of LPS 8,9. The high concentration of ruminal LPS overactivated the NF-κB and MAPKs inflammatory pathways in the rumen epithelium and partially induced rumenitis 10. Furthermore, LPS contribute to hindering the regeneration of epithelial cells and reduces the cell viability of rumen epithelial cells, potentially due to histamine (HIS) accumulation 11-13. This results in a disruption to the barrier function of the rumen epithelium, resulting in the translocation of LPS 14,15. This translocation allows LPS to enter the portal circulation and the peripheral circulation 16. LPS in the systemic circulation first binds to LPS-binding protein (LBP). It is then transported to the surface of immune cells, where it binds to CD14 to form the LPS-LBP-CD14 triple complex. It is then transported to the TLR4-MD2 protein complex, activating TLR4 and triggering an inflammatory response, such as rumenitis, mastitis, endometritis, laminitis, and hepatitis (Figure 1) 17-20. These diseases can result in substantial economic losses for the dairy industry, including expenses associated with reduction in milk fat and protein, forced culling of cows, treatment costs, milk production losses, veterinary fees, and expenditures on veterinary pharmaceuticals 12,21,22. Therefore, SARA exerts various adverse effects on dairy cows' health, ultimately limiting the progress of the dairy industry.

In recent years, the concept of “trained immunity” was introduced in the field of immunology 23. It is a non-specific immune response with “memory” characteristics that occurs in innate immune cells. This process differs from the differentiation of memory immune cells in adaptive immunity. Instead, it is associated with epigenetic reprogramming, metabolic reprogramming, and long-term protection mediated by hematopoietic stem cells 24. Trained immunity refers to the persistent changes in innate immune cell function induced by certain microbial stimuli and endogenous ligands, ultimately leading to heightened responses to subsequent challenges 25. Typically, β-glucan components of fungal cell walls or Bacillus Calmette-Guérin (BCG) vaccines are employed as an initial stimulus to induce trained immunity, which enhances host resistance to heterologous stimuli, including LPS and viruses 26. Indeed, the success of LPS in inducing trained immunity as an initial stimulus has also been demonstrated in studies of microglia 27.

Therefore, LPS plays a crucial role in both SARA and host immunity. In this review, we aim to establish the connection between SARA and trained immunity. Specifically, we present the latest insights into SARA, highlighting potential directions for future research in this field. Additionally, we examine the structural characteristics of LPS from various sources, analyzing and comparing their capacities to initiate immune responses. Importantly, the prevalence of LPS in the rumens of animals with SARA underpins the development of targeted strategies to mitigate both SARA and its associated inflammatory effects. Furthermore, we explore the theory, mechanisms of trained immunity in various animals, discussing ongoing investigations and its potential applications in preventing and controlling of SARA. Finally, we propose a theoretical model that elucidates the complex interplay between SARA, LPS, and trained immunity, offering a comprehensive framework to tackle related inflammatory diseases.

## SARA promotes the inflammatory response

**Figure 2:**
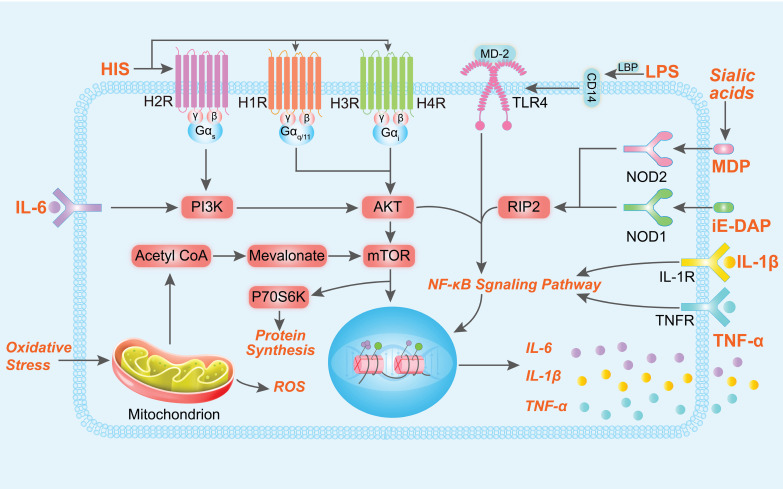
** Signaling pathways inflammation and oxidative stress induced by LPS, HIS, iE-DAP and MDP.** After binding to their respective receptors, LPS, HIS, iE-DAP, and MDP participate in the PI3K-AKT-mTOR and NF-κB signaling pathways, regulating gene expression and promoting the release of cytokines IL-1β, IL-6, and TNF-α. Mitochondria generate reactive oxygen species (ROS) in response to oxidative stress, and additionally, metabolic byproducts from mitochondrial metabolism can participate in the mTOR signaling pathway and regulate gene expression.

Animals with SARA typically exhibit reduced dry matter intake, decreased milk yield, and diminished milk fat and protein synthesis. Moreover, SARA has been associated with several inflammatory diseases, including rumenitis, mastitis, laminitis, and endometritis 10,12,13,28. During the pathogenesis of SARA-induced inflammatory disease, there is a notable increase in the levels of certain metabolites in the rumen, such as LPS, HIS, N-γ-d-glutamyl-meso-diaminopimelic acid (iE-DAP) and sialic acids (Figure 2) 4,29-32. The increase in LPS in the rumen fluid is closely related to the relative abundance of Proteobacteria and Bacteroidetes (such as *Escherichia coli*, *Succinivibrionaceae_UCG-001*,* Prevotella albensis*, *Prevotella brevis*, and* Prevotella ruminicola*) 9,33-35. These gram-negative bacteria undergo lysis in the ruminal low-pH environments, releasing cell wall-bound LPS and activating inflammatory responses through the TLR4/NF-κB signaling pathway 36. During SARA, the ruminal low-pH environments lead to dysbiosis of the rumen microbiota and increases in histamine-producing bacteria. HIS in the rumen is derived from *Allisonella histaminiformans*, which is able to grow in environments containing histidine and utilizes histidine as its only energy source 37. And HIS may participate in the PI3K-AKT-mTOR and NF-κB pathways by binding to HIS receptors 12,38. The release of inflammatory cytokines may also trigger the significant release of HIS from mast cells and basophils, exacerbating inflammatory reactions. This process may enhance neutrophil adhesion, partly through the inhibition of autophagy 39. In addition, rumen iE-DAP levels also increased significantly during SARA, which may be related to changes in Proteobacteria, *Prevotella*, and *Streptococcus bovis*, as these microbiota are susceptible to death and release large amounts of iE-DAP in low-pH environments 35,40-42. Studies have shown that iE-DAP can activate the NF-κB pathway by binding to NOD-like receptor 1 (NOD1) and induce inflammation and injury in bovine hepatocytes and mammary epithelial cells 43,44. It is worth noting that recent studies have identified the interaction of microbial derived muramyl dipeptide (MDP) with NOD-like receptor 2 (NOD2) as important factors in the pathogenesis of mastitis 45. Sialic acids (both Neu5Gc and Neu5Ac) is important differential metabolite in the rumen of healthy cows and SARA cows, which exacerbate mastitis in mice with gut dysbiosis 28. This gut dysbiosis facilitated *Enterococcus cecorum* expansion and promoted MDP release, which induced inflammation by activating the NOD2-RIP2-NF-κB axis 45. Additionally, elevated levels of multiple metabolites may increase the production of reactive oxygen species, leading to in oxidative stress and cellular damage 46,47.

Overall, the elevation of metabolites such as LPS, HIS, iE-DAP and MDP, and their associated inflammatory responses, is closely associated with the lysis of gram-negative bacteria in low-pH environments. Notably, the structure of LPS is recognized as a crucial factor influencing the efficacy of phage therapy. However, *E. coli* and its mutants express diverse LPS structures, resulting in varying infection phenotypes in phage therapy 48. Therefore, we aim to evaluate and elucidate the role of LPS in SARA-induced inflammatory responses, providing insights that may contribute to novel therapeutic strategies.

## The role of LPS in SARA-induced inflammatory responses

LPS have garnered initial interest, primarily for their ability to activate the immune system 49. However, due to structural and source variation among LPS molecules, host immune cells often exhibit specificity, responding primarily to certain serotypes of LPS 50. Notably, atypical sources of LPS may exert contrasting immunogenic or inhibitory effects on the same immune response 51,52. This underscores the need to investigate the structural and physiological characteristics of diverse LPS in the rumen of ruminants. Nevertheless, most LPS used in SARA studies originate from *E. coli* or LPS mixtures extracted from the rumen or digestive tract, with limited focus on specific bacterial sources that may have distinct immunogenic profiles 16,53-55.

**Figure 3:**
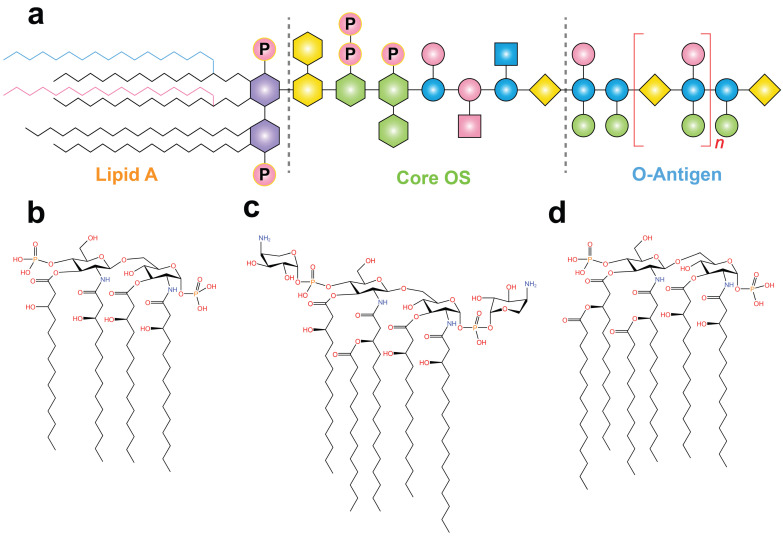
** The general chemical structure of LPS and the structural diversity in the lipid A region.** (**a**) The general chemical structure of LPS. The red and blue acyl chains represent two different acyltransferases encoded by genes *LpxL* and *LpxM*, respectively. These acyltransferases are responsible for the addition of fifth and sixth acyl chains to tetraacylated endotoxins. (**b**) Lipid IV_A_ consists of only four primary acyl chains. Lipid A derived from *Burkholderia cepacia* (**c**) and *E. coli* (**d**)*.* Adapted with permission from Di Lorenzo *et al.* 49. Copyright 2022, American Chemical Society.

LPS primarily consist of three distinct regions: lipid A, core oligosaccharide (OS), and O antigen (Figure 3). Lipid A is the central component of LPS, responsible for their endotoxicity and pyrogenic properties. The variation in the immune-stimulating potential of LPS has been linked to the strain and degree of lipid A acylation. Two acyltransferases, Kdo2-lipid IVA lauroyltransferase (*LpxL*) and lauroyl-Kdo2-lipid IVA myristoyltransferase (*LpxM*), add the fifth and sixth acyl chains to tetraacylated lipid A. Compared to tetraacylated and penta-acylated lipid A, hexa-acylated bis-phosphorylated lipid A exhibits the highest level of immune stimulation in mammals. Conversely, the presence of only four or five acyl chains in LPS inhibits the formation of TLR4/MD-2/LPS heterodimer formation and hinders signal transduction 56. Furthermore, given the absence of a comprehensive protocol for the detection of rumen LPS,* E. coli* hexa-acylated LPS is frequently employed in in vitro experiments on rumen tissue to study the inflammatory response of rumen acidosis 57. Therefore, further investigation into the diversity of LPS types and the degree of lipid A acylation in various biological samples, such as rumen fluid, serum, milk, and different tissues, is crucial for understanding their role in immune responses.

The O antigen is highly variable encompasses a wide range of serotypes. Although some serotypes of *E. coli* were eliminated, 181 O-antigens with different structures have been identified in this bacterial species 58. Additionally, bacteriophages bind to O antigens, enabling their attachment to bacterial core oligosaccharides and facilitating the infection of resistant bacterial strains 59. *E. coli* strains F5 and F17, and their mutants, exhibit different O antigens, resulting in varying levels of protection against phage infection 60,61. The O antigen also determines the sensitivity of *Pseudomonas protegens* to phage tail-like particles, and protects bacteria from the host immune system 62. However, there is a lack of targeted research on the role of LPS O antigen in SARA. Therefore, further exploration of the O antigen produced by the rumen core microbiota could lead to the development of targeted therapeutic strategies to against phage infections.

In contrast to the O-antigen, structural changes in the core OS region are relatively minor but can be further categorized into inner core and outer core OS modifications. Typically, the inner core OS contains 3-deoxy-D-*manno*-oct-2-ulosonic acid (Kdo), while the outer core exhibits more variability 63. Kdo is an eight-carbon sugar that connects lipid A to the core oligosaccharides 64. Therefore, differences in the core OS structure of LPS may affect the interaction between MD-2 and TLR4, as well as the homologous and heterologous dimerization of TLR4 56,65. Overall, lipid A, core OS, and O antigens play important roles in activating the immune system. The development of vaccines based on their antigenic characteristics is a potential approach for the prevention of related diseases. However, due to the limited development of precise LPS detection techniques, accurately predicting the source of different LPS strains based solely on their structure remains challenging.

The translocation of LPS into the systemic circulation disrupts homeostasis by altering the immune function of dairy cows. This ultimately leads to a pro-inflammatory state, which increases susceptibility to laminitis, ruminitis, endometritis and mastitis 31,33,66,67. Previous research has indicated a notably elevated concentration of LPS in various biological samples of ruminants prone to SARA or with SARA, including milk, mammary glands, lacteal veins, tail veins, feces and rumen fluids (Table 1). This observation suggested that LPS underwent translocation to these fluids. Similar findings were observed in an investigation on Hu sheep 31,46. Importantly, when LPS is absorbed into the peripheral blood circulation and reaches a specific concentration, the potential for endotoxemia subsequently arises 17. Subsequently, LPS and other toxins disseminate throughout the body, eliciting inflammatory responses in various tissues and organs 68. Low degree endotoxemia causes persistent damage and mastitis by activating the TLR4-cGAS-STING-NF-κB/NLRP3 signaling pathway. Furthermore, low-degree endotoxemia can also aggravate *E. coli* induced mouse mastitis by activating neuraminidase to damage host alkaline phosphatase 69. Similarly, the administration of LPS via intramammary injection results in the elevation of acute-phase proteins and modulation of the blood metabolome 70. LPS modulates the inflammatory response in the breast by activating the TLR4/NF-κB inflammatory signaling pathway, while simultaneously suppressing the synthesis of casein through the PI3K-AKT-mTOR signaling pathway 54,71. Thus, LPS not only circulates throughout the body but also negatively impacts dairy product quality.

Existing literature on the impact of LPS in SARA primarily focuses on total LPS levels in rumen fluid or serum. As mentioned earlier, validation tests often use LPS derived from a specific bacterium, particularly *E. coli*, to induce inflammation in cell or mouse models 85. However, it is crucial to recognize that LPS exhibits strain specificity. In the rumen, LPS is produced through the lysis of diverse gram-negative bacteria rather than being exclusively derived from *E. coli*. The varied sources of LPS can lead to distinct physiological effects, ranging from promoting to inhibiting inflammation 86. Early studies have shown that purified LPS from *E. coli*, *Salmonella typhimurium*, *Pseudomonas aeruginosa*, and *Klebsiella pneumoniae* exhibit different effects on the synthesis of inflammatory mediators such as TNF-α, IL-Iβ, IFN-γ, and IL-10. Recent research further suggests that *E. coli*-derived LPS can impair intestinal glucose absorption while enhancing insulin and glucagon-like peptide 1 secretion 86. In contrast, *Chlorella*-derived LPS has been shown to mitigate glucose abnormalities induced by equivalent doses of *E. coli* LPS, improve blood sugar regulation in obese mice, and promote beneficial endotoxemia 86. Similarly, *Salmonella*-derived LPS may induce a metabolically advantageous state of endotoxemia. These findings underscore the complexity of LPS effects, which cannot be fully captured by measuring endotoxin units or circulating LBPs alone.

Moreover, neutrophils have been found to distinguish between LPS from different bacterial species, such as* E. coli*, *Salmonella*, and *P. aeruginosa.* Interestingly, neutrophils respond specially to LPS derived from *E. coli* O128:B12 and *P. aeruginosa* 10, but not to other LPS types 87. This highlights the unique toxicity of *E. coli*-derived LPS compared to LPS from other common gram-negative ruminal bacteria, suggesting that *E. coli* LPS may not accurately represent the effects of ruminal LPS on the host 88-90. Recent studies also indicate that ruminal pH plays a role in determining LPS profiles. For instance, under low pH conditions, penta-acylated LPS is predominantly derived from *Prevotella*, whereas hexa-acylated LPS is mainly associated with *Succinivibrionaceae_UCG-001*, rather than *E. coli* 34. This finding underscores the necessity of comparing the virulence of common gram-negative ruminal bacteria, such as *Prevotella* and *Succinivibrionaceae_UCG-001*, with *E. coli* LPS. The microbial sources of LPS likely influence host responses in different ways during SARA. In summary, investigating the mechanisms of LPS derived from specific bacterial sources may provide valuable insights into systemic inflammation caused by SARA in dairy cows. Identifying beneficial LPS variants could further contribute to preventing inflammatory diseases, developing vaccines, improving milk quality, and fostering the healthy growth of the dairy industry.

## Trained immunity represents a type of congenital immunological memory

Animal immunity consists of two major components: innate immunity and adaptive immunity. Traditionally, innate immunity has been regarded as lacking the capacity for memory, in contrast to adaptive immunity. However, the conventional understanding that these systems differ solely based on the presence or absence of immune memory has been increasingly challenged 91. The concept of “trained immunity”, introduced in 2011, describes an intrinsic form of innate immune memory 23. Notably, trained immunity was first identified in organisms that rely exclusively on innate immunity, such as plants, invertebrates, and bacteria 92,93. These groups collectively represent the overwhelming majority of Earth's species diversity, comprising approximately 97-99% of all species 94. In contrast, vertebrates, which account for only 1-3% of total species diversity, possess both innate and adaptive immune systems, enabling them to develop immune memory through adaptive processes. Interestingly, evidence from plants challenges the traditional distinction between innate and adaptive immunity. When plants are infected by microbial pathogens, they develop enhanced systemic immunity against subsequent infections, effectively forming a “memory” of the initial encounter, despite lacking adaptive immunity 95. This demonstrates that the innate immune systems of plants possess memory-like capabilities, suggesting that similar mechanisms may also exist within the innate immune systems of animals.

**Figure 4:**
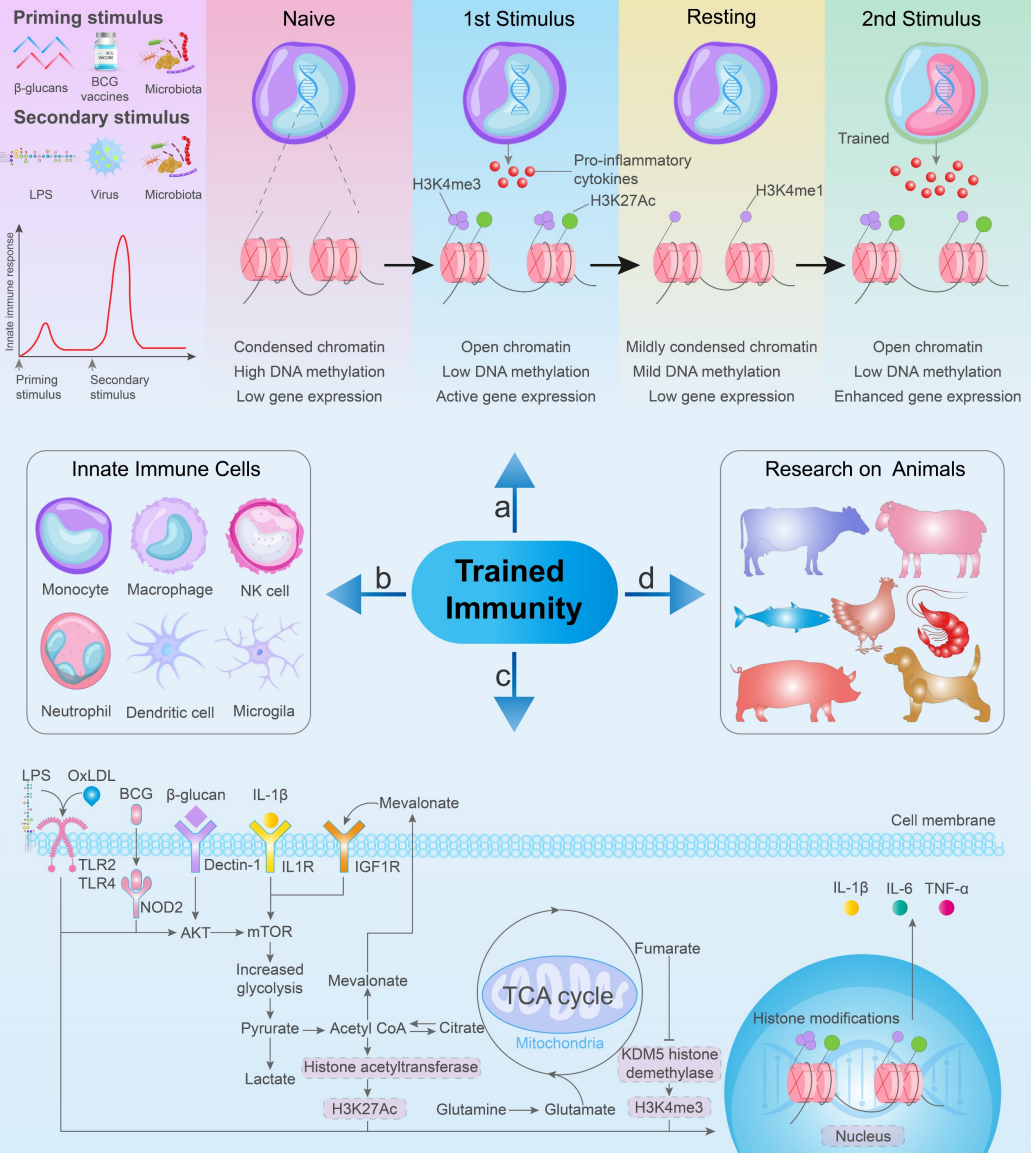
** The mechanism of trained immunity and related research.** (**a**) Priming stimulus and secondary stimulus for trained immunity. Following the initial stimulation, innate immune cells undergo epigenetic modifications and metabolic reprogramming, releasing a small amount of pro-inflammatory cytokines. Subsequent heterologous stimulation leads to an enhanced immune response and an increased release of pro-inflammatory cytokines. (**b**) Specific innate immune cells contribute to trained immunity. (**c**) Common metabolic and epigenetic pathways in trained immunity involve the activation of the mTOR signaling pathway and subsequent glycolysis, initiated by BCG vaccine, β-Glucan, and OxLDL. (**d**) Trained immunity in animals.

Early evidence supporting innate immune memory can be found in a study conducted in the 1960s, which indicated that mice that received the BCG vaccine exhibited protection against subsequent infections caused by *Listeria monocytogenes* and *S. Typhimurium* 96. Following the initial immune response, innate immune cells transition to a resting state. However, through epigenetic modifications, these cells can exhibit increased inflammation and antimicrobial properties upon re-exposure to antigens (Figure 4a) 94,97. This phenomenon results in an enhanced non-specific response to subsequent infections, ultimately improving the survival rate of the host 91,98,99. Currently, the initial stimuli employed for trained immunity primarily include BCG, β-glucan, recombinant proteins, cytokines, and peptidoglycan. Similarly, the secondary stimuli for trained immunity mainly include LPS, viruses, bacteria, and β-glucan (Figure 4a) 26,100. Numerous studies have revealed the distinct features of innate immune cells, such as monocytes, macrophages, natural killer cells, dendritic cells, neutrophils, and microglia, that play crucial roles in trained immunity (Figure 4b) 27,97,101-104. At the cellular level, different stimuli can induce epigenetic changes in response to TLR agonists, affecting microbe clearance and inflammatory cytokine secretion in *in vitro*. Notably, this process is requiring glycolysis, mevalonate synthesis, and mTOR activation (Figure 4c).

Research into trained immunity has progressed through various animal experiments (Figure 4d). In a study involving laying hens and broiler chickens, trained immunity of monocytes could be induced by β-glucan, followed by LPS stimulation, leading to increased mRNA levels of IL-1β 105,106. Similar findings were reported in dogs, where the synthesis of pro-inflammatory and antimicrobial compounds was triggered in monocytes following bacterial stimulation after β-glucan induction 107. Similarly, evidence of trained immunity has also been identified in bony fish and shrimp 108-111. Nonetheless, immune training research on pigs has yielded varied results; the use of the BCG vaccine or β-glucan to induce trained immunity in pig monocytes can lead to either a training or tolerance state 112. However, administration of the BCG vaccine did not restrict the replication of swine influenza A virus or prevent clinical symptoms in this pig model 113. Alternatively, heat-inactivated *Mycobacterium bovis* immune stimulators have been found to enhance non-specific protection against *Salmonella cholera* infections in pigs 114. This research provides evidence for trained immunity not only in humans and mice, but also in domesticated animals, including ruminants. Newborn goats and calves can exhibit activated trained immunity following the oral administration of yeast-derived β-glucan, which enhances their resistance to subsequent LPS stimulation 115,116. Studies have also shown that milk and its component, bovine immunoglobulin G (bIgG), may trigger trained immune responses in human monocytes. When re-stimulated with the TLR1/2 ligand, Pam3CSK4, monocytes previously exposed to raw milk exhibited increased IL-6 production compared to non-trained monocytes. Moreover, following stimulation with TLR4 and TLR7/8, prior bIgG training led to increased cytokine production 102,117. Nonetheless, other milk components may also activate trained immunity 118. Therefore, investigating whether raw milk and bIgG can elicit trained immunity in bovine monocytes and macrophages represents a promising research direction. Additionally, aerosol BCG vaccination of young calves has been found to induce a "trained" phenotype in circulating PBMCs in *in vivo* assays. This induction of the trained phenotype was linked to increased Toll-like receptor expression compared to that in PBMCs from unvaccinated control calves. Furthermore, the vaccinated calves exhibited significantly enhanced pro-inflammatory cytokine responses and metabolic reprogramming 119.

## SARA and trained immunity

The concept of trained immunity has deepened our understanding of the role of innate immunity in preventing infection and inflammation. Furthermore, this phenomenon has heightened interest in the correlation between innate and adaptive immunity and their intricate interplay to combating infection and inflammation. LPS plays a key role in the SARA-induced inflammatory response as well as the activation of trained immunity. This observation highlights the importance of exploring the relationship between SARA and trained immunity. However, there are currently no reports on the association between SARA and trained immunity. This review aims to identify potential connections between SARA and trained immunity based on existing studies.

The use of *Saccharomyces cerevisiae* as a feed additive has been associated with the development of trained immunity. *S. cerevisiae* effectively enhances the growth performance, including weight gain and feed efficiency, in pigs, chickens, and calves 120-122. It has also been shown to reduce greenhouse gas emissions from ruminants, including carbon dioxide and methane 123,124. These beneficial effects may be linked to the β-glucans abundant in the cell wall of *S. cerevisiae*, owing to their immunomodulatory and physiological functions. Notably, supplementation *S. cerevisiae* and β-glucan has been found to enhance immune responses in chickens against non-specific pathogens, such as *Salmonella* and Newcastle disease 122,125. This observation suggests that β-glucan likely serve as a primary stimulus for activating trained immunity in these animals.

In a study of newborn goats, researchers administered the probiotic *Debaryomyces hansenii* containing β-glucan orally. This enhanced cellular phagocytosis, nitric oxide production and glycolysis, glucose consumption and lactate production 126. After LPS stimulation, plasma levels of IL-1β, IL-6 and TNF-α increased, which indicated the successful induction of trained immunity 115. As a common feed additive for ruminants, the effect of *S. cerevisiae* containing β-glucan on cows with SARA have been widely studied. These effects include benefits such as increased feeding frequency, milk fat production, anti-inflammatory activity, and elevated rumen pH 127-129. Furthermore, *S. cerevisiae* has been shown to enhance adaptive immunity, reduces free LPS concentration, and lowers serum IL-1β levels in cows with SARA 72. Specifically, supplementation with yeast culture can enhance the activation of innate immune responses in neutrophils. This results in increased glucose utilization through enhanced oxidative bursts in these cells 130. According to previous reports, neutrophils exposed to β-glucan training may also exhibit anti-tumor effects 131. A recent study found that neutrophils trained with *Shigella* exhibit increased bacterial clearance efficiency, although this training differs from BCG- and β-glucan-induced training 132,133. These observations may provide a mechanistic explanation for the inhibitory effect of *S. cerevisiae* on rumen LPS concentrations in cows with SARA. Therefore, activating trained immunity in cows via β-glucan may help to mitigate the inflammatory responses associated with SARA.

In addition, findings from studies on trained immunity in calves may also offer valuable insights. Guerra-Maupome *et al.* and Samuel *et al.* induced trained immunity in calves using aerosolized BCG and subcutaneous BCG injections, respectively, which was reflected in the enhanced production of pro-inflammatory cytokines following LPS stimulation 119,134. In vitro studies have shown that β-glucan upregulates the expression of co-stimulatory molecules CD80 and CD86 on the surface of bovine monocytes. Moreover, stimulated cells showed increased IL-8 production and mRNA expression of TNF-α, IL-1β and IL-6 in a dose-dependent manner, indicating evidence for trained immunity in bovine monocytes 135. However, no relevant research has been conducted on trained immunity in cows with SARA. In cows, SARA is often characterized by reduced ruminal pH and elevated levels of LPS, HIS, and iE-DAP. Elevated levels of TNF-α, IL-1β, and IL-6 have been detected in the milk, rumen fluid, and blood of cows with SARA 10,33,39,73. These inflammatory cytokines play a crucial role in mediating the inflammatory response of the rumen epithelium through paracrine signaling. This process partially contributes to ruminal inflammation in cows with SARA. Elevated levels of LPS, HIS, iE-DAP and MDP in the rumen and other tissues are closely associated with increased pro-inflammatory factor production and the activation of inflammatory signaling pathways.

The AKT-mTOR signaling pathway plays a crucial role in the activation of trained immunity. BCG, oxLDL, and β-glucan, as the initial stimuli for trained immunity, can engage the AKT-mTOR signaling pathway by binding to NOD2, TLR2/TLR4, and Dectin1 receptors, respectively 136. Interestingly, LPS, HIS, iE-DAP, and MDP can also bind to receptors such as TLR4, HIS receptors, and NOD1/NOD2, activating the AKT-mTOR and NF-κB signaling pathways. These pathways further trigger a series of epigenetic modifications, including histone modifications and DNA methylation, ultimately leading to changes in gene expression patterns 91,98. During SARA, elevated levels of stimuli such as LPS, HIS, iE-DAP, and MDP may prompt trained immune cells to mount a faster and more robust response to these challenges, thereby accelerating the initiation of inflammation and driving the secretion of increased amounts of pro-inflammatory cytokines 91,98. Overall, BCG and β-glucan may activate trained immunity in SARA cows, enhancing non-specific immune responses to LPS from various sources. This activation may help reduce the occurrence of inflammatory reactions. However, further research is necessary to confirm the underlying mechanisms.

A growing body of research shows that LPS exposure can activate trained immunity and enhance the protective functions of innate immune cells against heterologous stimuli. A recent study demonstrated that a single low-dose exposure of the respiratory mucosa to LPS induces sustained changes in the immune phenotype of airway macrophages, promoting trained immunity and providing robust protection against acute pneumococcal infections 137. LPS exposure can also induce profound and long-lasting remodeling of signaling pathways involved in response to LPS or fungal pathogens. Subsequent exposure to LPS or fungal stimuli may lead to immune tolerance or immune training phenotypes, respectively, depending on the type of secondary stimulus 138. Thus, given the diverse sources of LPS in the rumen of cows with SARA, certain LPS variants might beneficially activate innate immunity and enhance resistance to toxic LPS. While previous studies have explored the beneficial and toxic effects of LPS from various bacterial sources, research specifically examining the impact of different LPS molecules on SARA remains limited. This raises questions about which bacteria produce beneficial LPS and the role these molecules play in trained immunity. Ultimately, this underscores the need to identify and compare the bacterial LPS sources in cows with SARA and healthy cows.

The induction of trained immunity is closely associated with epigenetic modifications, including changes in chromatin structure and gene expression. This corresponds to the suppression of inflammatory genes during LPS tolerance, allowing for maintained or enhanced expression of genes involved in antibacterial responses 24,139. Therefore, it is crucial to elucidate the role of LPS in SARA and trained immunity activation. Additionally, investigating how pro-inflammatory factors function within the interplay of innate and adaptive immunity during SARA could advance our understanding of immune training and adaptive responses.

The memory duration of trained immunity is notably shorter than that of classical adaptive immunological memory 24. Different initial stimuli can exert either beneficial or detrimental effects on immune cells, depending on the context and duration of exposure 140. SARA in dairy cows primarily occurs during the perinatal and peak lactation periods, with minimal occurrence at other stages. Thus, the transient nature of this prevention strategy corresponds well with the elevated incidence of SARA during these stages. Effective treatment strategies for trained immunity require precise targeting of immune-related molecular mechanisms in select cells within a limited activation window 136. It is crucial to explore the mechanisms linking SARA and trained immunity, particularly the roles of specific contributing factors at different physiological stages. Prolonged stimulation of mammalian bone marrow progenitor cells has been shown to generate “trained” myelocytes, providing a potential basis for long-term therapeutic interventions 98.

## Preventative measures for SARA

To mitigate the economic losses caused by SARA on commercial farms, it is essential to implement effective preventive and corrective measures. Currently, commercial farms have adopted the total mixed rations feeding model, reducing the reliance on high-corn or high-wheat diets. This approach effectively mitigates selective feeding and excessive grain consumption, significantly reducing the occurrence of SARA 141,142. Furthermore, maintaining a clean and well-ventilated environment alleviates stress levels associated with SARA. Recent studies show that sodium bicarbonate reduces biogenic amines and iE-DAP in the rumen, leading to an increase in rumen pH and improved microbial ecology. Sodium bicarbonate consumption may also alleviate mastitis in ruminants through the NOD1/RIRP2 signaling pathway 18,143,144.

Plant extracts contain bioactive compounds with antioxidant, antibiotic, antiviral, anticancer, antiparasitic, and antifungal properties 145,146. Extracts derived from sources such as green tea, chestnut shells, bamboo leaves, and grape pomace have been shown to benefit animal intestinal flora, reduce oxidative stress, modulate inflammatory reactions, and enhance immune function 147-152. Consuming a combination of naringin, glycyrrhetinic acid, cinnamon extract, and other plant extracts has been shown to increase rumen pH, improve the bacterial community, and suppress inflammatory reactions, effectively managing rumen acidosis 153-156. Most of these plant extracts are flavonoids or polyphenols. Flavonoids are believed to act as epigenetic modulators, mediating changes and participating in disease prevention and treatment 157. Curcumin, an important polyphenolic compound, has been shown to boost anti-inflammatory, antioxidant, and immune capabilities in post-weaning cows, although its role in SARA cows remains under-researched 158. Curcumin may act as an epigenetic regulator of histone acetyltransferases and modifications, contributing to its involvement in trained immunity 159. Resveratrol, another polyphenolic compound, is believed to increase beneficial rumen bacteria, reduce methanogenic bacteria, and promote average daily weight gain in fattening goats 160. Resveratrol also induce various chemical modifications, such as oxidation, dihydroxylation, and demethylation, enhancing the ability of BCG to induce trained immunity 161,162. Future studies should explore a broader range of plant extracts and their potential combination with trained immunity research to improve SARA treatment efficacy. This approach not only maximizes the utilization of plant residues but also helps commercial farms achieve cost savings, improve energy efficiency, and enhance overall performance.

*S. cerevisiae*, *Lactobacilli* spp., rumen-derived *Diutina rugosa*, and multi-strain probiotics have been shown to stabilize or increase rumen pH, regulate the intestinal microbiota, and enhance the population of cellulose-degrading microorganisms in the rumen 72,163-167. Of particular significance is that *S. cerevisiae*, *Lactobacilli* spp. and *Diutina rugosa* all contain β-glucan. This suggests that some β-glucan-containing probiotics may exert their beneficial effects by stimulating trained immunity, thereby reducing rumen LPS concentrations and inflammation 74. *Akkermansia muciniphila* is a promising probiotic that protect gut barrier function, reduce inflammatory cytokine levels, and has potential to induce trained immunity and mitigate inflammation 168.

However, its role in the relationship between SARA and trained immunity requires further research. Another study showed that *Lactococcus lactis* can induce innate immune memory and improve cure rates in cows with chronic mastitis 169. In conclusion, there is strong evidence suggesting that probiotics can significantly enhance the health and well-being of ruminant animals. However, there is currently no direct evidence that probiotics reduce SARA incidence by activating trained immunity.

Thiamine deficiency is a significant risk factor in lactic acidosis and alcohol use disorder 170,171. Both animals and humans rely on dietary thiamine 172. Thiamine supplementation has been shown to increase the abundance of microorganisms and enzymes that break down carbohydrates, while inhibiting lactic acid production 173-176. Furthermore, thiamine supplementation regulates the Nrf2/NF-κB signaling pathway, enhancing rumen epithelial barrier function. It also improves the structure of intestinal tissue and the microbial environment 124,177,178. As a result, numerous studies show that thiamine supplementation improves ruminal health by enhancing the microbiota composition and regulating inflammatory signaling pathways.

In summary, several strategies are available to increase ruminal pH and improve ruminal health. Notably, these include dietary supplementation with sodium bicarbonate, probiotics, plant extracts, and thiamine. However, SARA in ruminants primarily cause systemic inflammation, tissue damage, and a reduced milk production and quality. Thus, we propose that the combining strain-specific LPS with trained immunity induction may offer a novel approach to limit systemic inflammation.

## Future directions and conclusions

**Figure 5:**
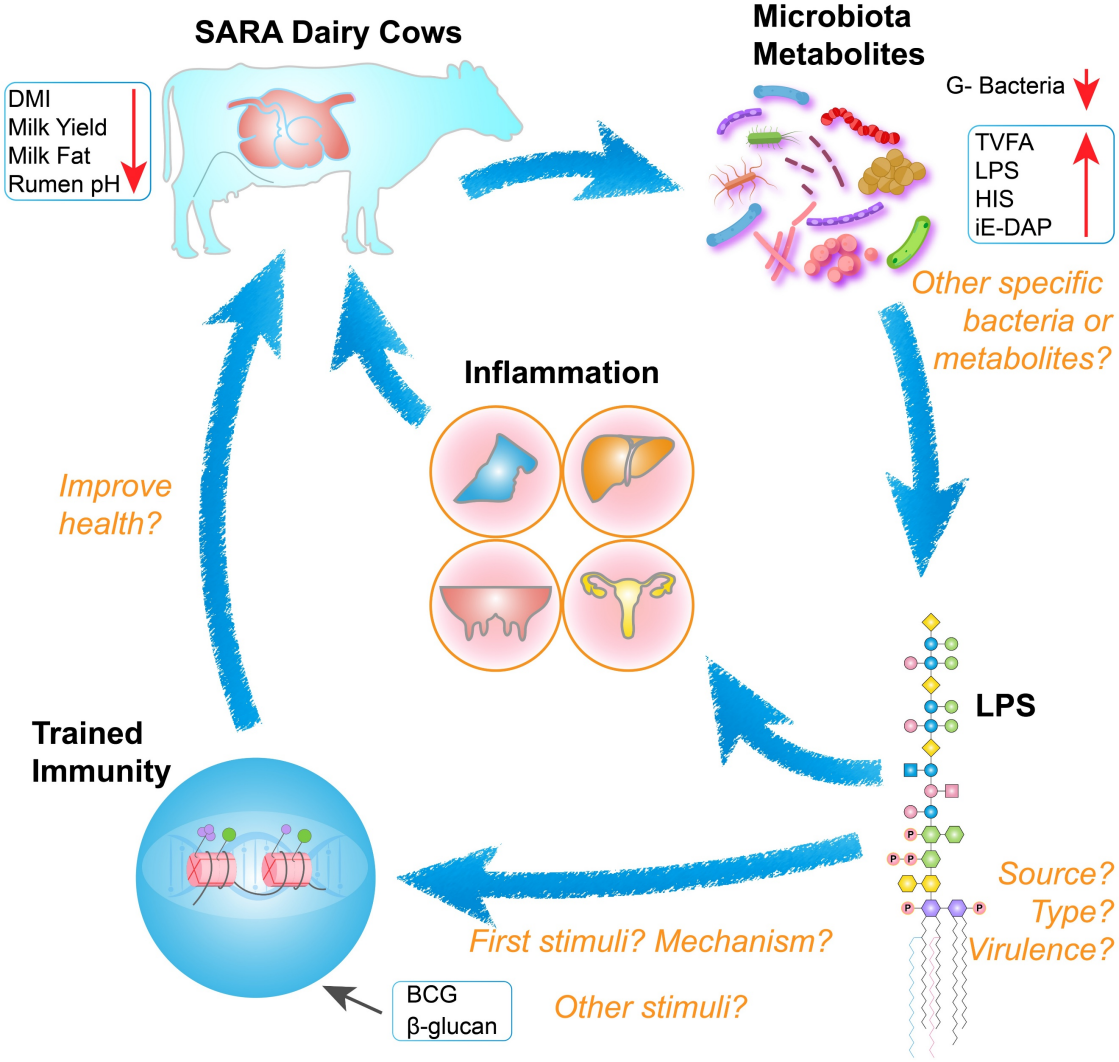
** The interrelationships between SARA, LPS, and trained immunity, alongside key research considerations.** Cows with SARA exhibit reduced dry matter intake, diminished milk production, milk fat depression, and decreased rumen pH. Decreased rumen pH leads to disruption of microbial structures and an increase in abnormal metabolites. Elevated levels of LPS in the systemic circulation can cause inflammation and adversely affect cow health. Different types of LPS may be involved as initial stimuli or secondary stimuli in the activation of trained immunity, ultimately improving the health of SARA cows.

SARA contributes to the onset of inflammatory diseases, particularly by disrupting rumen microbiota. This disruption can lead to mastitis, posing a significant threat to the quality and safety of dairy products 28,179. HC diets increase the abundance of starch-degrading bacteria, leading to a drop in rumen pH in dairy cows. In low pH environments, gram-negative bacteria undergo lysis, releasing metabolites such as LPS, HIS, and iE-DAP. These metabolites subsequently enter peripheral blood circulation and trigger an inflammatory response. While many studies have examined SARA's occurrence and prevention, few have explored its links to trained immunity. However, the beneficial effects of *S. cerevisiae* and β-glucan in mitigating SARA and reducing LPS levels offer valuable insights 117. To our knowledge, this is the first review to explore the potential links between SARA, LPS, and trained immunity (Figure 5).

HC diets are commonly used in research to induce SARA for study purposes. With advancements in commercial ranching management and implementation of effective techniques, the overall well-being of cows has significantly improved. However, variations in the incidence of SARA can still occur even when following the same diet 180. This variability has been linked to differences in fecal bacteria and fatty acid profiles in pre- and postpartum cows during the perinatal period 181. Early-lactating Simmental cows also exhibit varying susceptibility to SARA even on identical grain-rich diets, highlighting that dietary factors are not the sole contributors to SARA in ruminants 182. Interestingly, studies on SARA susceptibility in cows and sheep revealed distinct rumen microbiota compositions. SARA-tolerant animals exhibited a higher proportion of fiber-degrading bacteria, while SARA-susceptible groups had more starch-degrading bacteria 183,184. This indicates that the balance between fiber- and starch-degrading bacteria is a key determinant of SARA susceptibility and may characterize SARA-tolerant cows. Future research should explore non-dietary factors and further characterize SARA-tolerant cows.

With advancements in omics technologies, the integration of multi-omics approaches has become a powerful strategy for accurately predicting the diagnosis, prognosis, and treatment of various diseases 185. A notable shift in research focus from single-omics to multi-omics analyses has further enhanced this potential 28,186. The combined analysis of the microbiome, transcriptome, metabolome, and metagenome has greatly advanced the study of SARA, providing deeper insights into its underlying mechanisms. In particular, these approaches have elucidated the molecular interactions between rumen bacterial communities and their host under HC diet conditions, paving the way for novel molecular regulatory strategies to mitigate the adverse effects of SARA on ruminants.

The activation of animal innate immunity using microbial-derived LPS represents a promising therapeutic approach. LPS from intestinal *Bacteroides,* which is less potent and more antagonistic than the highly inflammatory LPS by* E. coli*, may offer reduced efficacy in suppressing inflammatory processes 187. Specifically, low-acylated lipid A, a component of LPS, has the ability to inhibit TLR4 signaling by competing with high-acylated lipid A, thereby mitigating inflammatory responses 49. As essential glycolipids, LPS molecules play critical roles in biological processes across diverse organisms. Currently, various techniques are employed to detect LPS, including the rabbit pyrogen test, limulus amebocyte lysate assay, antibody- and aptamer-based biosensors, endotoxin activity assays, and single-molecule nanopore analysis 188-191. These methods enable the qualitative and quantitative analysis of LPS in biological samples. However, identifying the precise structure and acylation patterns of individual LPS molecules within complex samples remains a significant challenge. Antibody-based biosensors, while offering sensitivity and rapid detection, are costly and time-intensive 189. The integration of omics technologies has facilitated advancements in LPS detection analysis. By combining these approaches, researchers can gain deeper insights into the structural diversity and biological functions of LPS molecules in rumen fluid, blood, and feces from cows with SARA. This knowledge will support the creation of an LPS database to identify corresponding LPS-producing microorganisms. Developing this database requires elucidating the relationships between microbial LPS in dairy cows, characterizing structural variations in these molecules, and understanding their recognition by the host immune system. Such efforts could significantly enhance our comprehension of the role of LPS in health and disease.

Common stimuli for inducing trained immunity include the BCG vaccine and β-glucan. Recent studies have expanded this list to include non-classical stimuli such as inactivated *vitiligo syndrome virus*, inactivated *Mycobacterium bovis*, nanodrugs, and the COVID-19 adenovirus vaccine 97,192-195. The discoveries mark a significant advancement in immunology, offering innovative strategies for preventing and treating various diseases. In the context of SARA, yeast-derived β-glucan has shown promising potential to enhance immune responses in affected cows, suggesting its utility as a targeted intervention. However, further studies are required to elucidate the mechanisms underpinning immune training in cows, particularly regarding epigenetic modifications and the activation of innate immune cells. The identification of additional non-classical inducers of trained immunity opens a fascinating avenue for research, with potential applications in both veterinary and human medicine. Investigating their effects and mechanisms will likely uncover novel therapeutic strategies to combat inflammation and improve health outcomes.

Recently, numerous studies have elucidated the mechanisms through which SARA induces inflammation. The application of multi-omics technologies shows great promise in identifying targeted therapeutic drugs and addressing the challenges of current SARA treatment strategies. Emerging trained immunity provides new perspectives for comprehensively investigating SARA-related issues, particularly by examining the roles of microorganisms, their metabolites, and host immunity. Specifically, this can be achieved by considering the roles of microorganisms, their metabolites, and host immunity. Future research should integrate studies on SARA, multi-omics approaches, LPS, and immune system interaction networks to advance animal health. These findings could support the sustainable development of the dairy industry and ultimately enhance human well-being.

## Figures and Tables

**Table 1:** LPS levels in SARA and SARA susceptible ruminants.

Animal	Rumen fluid	Blood	Milk	Gut or feces	Tissues	Reference
Dairy cows	Control group: 30,768 ± 1035 EU/mL SARA group: 130,589 ± 1675 EU/mL ↑	Control group: <0.005 EU/mL SARA group: 0.21 ± 0.06 EU/mL ↑	-	-	-	10
Dairy cows	-	Hepatic vein and portal vein ↑	-	-	-	16
Dairy cows	Control (LC) group: 47,170 EU/mL SARA (HC) group: 79,040 EU/mL ↑	Jugular vein: Control (LC) group: 470 EU/mL SARA (HC) group: 860 EU/mL ↑	-	-	-	19
Dairy cows	-	Mammary veins ↑	-	-	-	22
Dairy cows	-	↑	-	-	-	29
Dairy cows	Control group: 19.7 EU/mL SARA (HC) group: 26.8 EU/mL ↑	Control group: 12.0 EU/mL SARA (HC) group: 17.0 EU/mL ↑	-	-	-	30
Sheep	↑	↑	-	-	Uterus ↑	31
Dairy cows	↑	Tail vein and lacteal veins ↑	↑	-	Mammary gland ↑	33
Dairy cows	-	↑	-	-	-	34
Dairy cows	Control group: 25,704 - 29,492 EU/mL SARA group: 73,283 - 151,985 EU/mL ↑	Control group: <0.05 EU/mL SARA group: 0.31 - 0.81 EU/mL ↑	-	-	-	35
Sheep	↑	↑	-	-	-	46
Dairy cows	Control (LG) group: 47.17 × 10^3^ EU/mL SARA (HC) group: 79.04 × 10^3^ EU/mL ↑	Hepatic vein, portal vein and jugular vein ↑	-	-	-	53
Dairy cows	↑	-	-	Feces ↑	-	72
Goats	Control group: 26.46 × 10^3^ EU/mL SARA (HC) group: 48.37 × 10^3^ EU/mL ↑	Control group: 0.28 EU/mL SARA (HC) group: 0.69 EU/mL ↑	-	-	-	73
Dairy cows	↑	-	-	Cecum and feces ↑	-	74
Dairy cows	-	Control (LC) group: 7.17 ± 1.25 μg/mL SARA (HC) group: 12.12 ± 1.26 μg/mL ↑	-	-	-	75
Dairy cows	↑	-	-	-	-	76
Dairy cows	↑	No significance	-	Cecal digesta and feces ↑	-	77
Dairy cows	↑	-	-	-	-	78
Dairy cows	Control (LC) group: 4.921 × 10^5^ EU/mL SARA (HC) group: 7.855 × 10^5^ EU/mL ↑	Portal vein: Control (LC) group: 0.106 EU/mL SARA (HC) group: 0.204 EU/mL ↑	-	Cecum content: Control (LC) group: 11.960 × 10^5^ EU/g SARA (HC) group: 13.115 × 10^5^ EU/g ↑	-	79
Goats	-	-	-	Colon digesta ↑	-	80
Goats	Control group: 15.38 × 10^3^ EU/mL SARA group: 21.81 × 10^3^ EU/mL ↑	Control group: 0.18 × 10^3^ EU/mL SARA group: 0.54 × 10^3^ EU/mL ↑	-	-	-	81
Goats	-	↑	-	-	-	82
Sheep	↑	Hepatic vein ↑	-	-	-	83
Sheep	↑	Hepatic vein and portal vein ↑	-	colon content ↑	-	84

## Abbreviations

BCG: Bacillus Calmette-Guerin vaccine
bIgG: bovine immunoglobulin G
HC: high-concentrate
HIS: histamine
iE-DAP: N-γ-d-glutamyl-meso-diaminopimelic acid
IFN-γ: interferon γ
IL-1β: interleukin 1β
IL-6: interleukin 6
LBP: lipopolysaccharide binding proteins
LC: low-concentrate
LPS: lipopolysaccharide
*LpxL*: kdo2-lipid IVA lauroyltransferase
*LpxM*: lauroyl-Kdo2-lipid IVA myristoyltransferase
MAPK: mitogen-activated protein kinases
MD-2: myeloid differentiation factor-2
MDP: muramyl dipeptide
mTOR: mechanistic target of rapamycin
NF-κB: nuclear factor-kappa B
NOD1/2: NOD-like receptor 1/2
Nrf2: nuclear factor erythroid 2-related factor 2
OS: oligosaccharide
PBMC: peripheral blood mononuclear cells
PI3K: phosphatidylinositol 3-kinase
ROS: reactive oxygen species
SARA: subacute ruminal acidosis
TLR2/4: toll-like receptor 2/4
TNF-α: tumor necrosis factor α
VFAs: volatile fatty acids
